# Changes in body core and body surface temperatures during prolonged swimming in water of 10°C—a case report

**DOI:** 10.1186/2046-7648-1-8

**Published:** 2012-11-01

**Authors:** Christoph Alexander Rüst, Beat Knechtle, Thomas Rosemann

**Affiliations:** 1 Institute of General Practice and for Health Services Research, University of Zurich, Zurich, Switzerland; 2 Gesundheitszentrum St. Gallen, Vadianstrasse 26, St. Gallen, 9001, Switzerland

**Keywords:** Hypothermia, Afterdrop, Body fat, Skinfold thickness, Open-water swimming

## Abstract

**Background:**

This case report describes an experienced open-water ultra-endurance athlete swimming in water of 9.9°C for 6 h and 2 min.

**Methods:**

Before the swim, anthropometric characteristics such as body mass, body height, skinfold thicknesses, and body fat were determined. During and after the swim, body core (rectum) and body surface (forearm and calf) temperatures were continuously recorded.

**Results:**

The swimmer (53 years old, 110.5 kg body mass, 1.76 m body height, 34.9% body fat, and a body mass index of 35.7 kg/m^2^) achieved a total distance of 15 km while swimming at a mean speed of 2.48 km/h, equal to 0.69 m/s, in water of 9.9°C. Body core temperature was at 37.8°C before the swim, increased to a maximum of 38.1°C after approximately 20 min of swimming, and then decreased continuously to 36.3°C upon finishing the swim. The lowest body core temperature was 36.0°C between 35 and 60 min after finishing the swim. Sixty minutes after the swim, the body core temperature continuously rose to 36.5°C where it remained. At the forearm, the temperature dropped to 19.6°C after approximately 36 min of swimming and decreased to 19.4°C by the end of the swim. The lowest temperature at the forearm was 17.6°C measured at approximately 47 min before the athlete stopped swimming. At the calf, the temperature dropped to 13.0°C after approximately 24 min of swimming and decreased to 11.9°C at the end of the swim. The lowest temperature measured at the calf was 11.1°C approximately 108 min after the start. In both the forearm and the calf, the skin temperature continuously increased after the swim.

**Conclusions:**

This case report shows that (1) it is possible to swim for 6 h in water of 9.9°C and that (2) the athlete did not suffer from hypothermia under these circumstances. The high body mass index, high body fat, previous experience, and specific preparation of the swimmer are the most probable explanations for these findings.

## Background

Athletes strive to push the limits of human performance. In indoor pool swimming, athletes try to swim as fast as possible [1] or to achieve as many kilometers as possible in ultra-swimming [2]. Another challenge for open-water swimmers is to swim as long as possible in cold water [3-9]. To date, the lowest reported water temperature in a scientific study in open-water swimming was 4°C where a swimmer was able to achieve a swim distance of 2.2 km within 42 min under this condition [7]. In 1969, Keatinge et al. [5] reported that two men were able to swim in water of 4.7°C for 1.5 and 7.6 min, respectively. At higher temperatures, swimmers were able to stay longer in open water. In the ‘New Year's Day Alcatraz Swim’, 11 swimmers were able to complete the 3-km swim between 31.3 and 62.6 min while swimming in 11.7°C cold water [8]. In an experiment, ten volunteers undertook three self-paced breaststroke swims in water at 25°C, 18°C, and 10°C where one swimmer had to quit after 61 min and four swimmers stopped before 90 min [9]. In a 92-km relay swim in Finnish lakes, eight swimmers experienced cold water swimming and achieved a mean swim time of 52.3 min in water with a mean temperature of 10.2°C [6]. In 1955, Pugh and Edholm reported from a swimming race across the English Channel where 18 of 20 participants completed the distance between France and England within 12 to 20 h in water of 15.5°C [10]. This was an interesting finding since records of shipwreck survivors showed that men can survive in the ocean at 15.5°C only for about 5 h [11]. Tolerance to water at temperatures below 20°C is limited by the loss of body heat at a rate which exceeds heat production. The internal temperature gradient cannot be maintained, and the rectal temperature falls at a rate which becomes increasingly faster, the lower the water temperature [10,11].

We report the case of an open-water ultra-swimmer who was able to achieve a swim distance of 15 km within 6 h and 2 min while swimming at a speed of 2.48 km/h in water of 9.9°C. Body core (rectum) and body surface temperature at the extremities (forearm and calf) were continuously recorded. The swimmer intended to swim as long as possible in water of 9.9°C, but based upon existing literature, we hypothesized that (1) he would not be able to swim for longer than approximately 60 min and that (2) he would suffer from both severe hypothermia and afterdrop as has been described in athletes swimming in warmer water [3,8].

## Case presentation

### Subject

Our subject was an experienced open-water ultra swimmer (53 years old, 110.5 kg body mass, 1.76 m body height, and a BMI of 35.7 kg/m^2^). The experiment was approved by the ethical committee of the Kanton, St. Gallen, Switzerland. The swimmer has a broad experience in open-water ultra-swimming. He completed in 2009 the 26.4-km swim in Lake Zurich within 11 h and 1 min. In 2010, he crossed the English Channel in a team relay. In July 2011, he crossed the Great Belt between Germany and Denmark, swimming from Germany to Denmark and back to Germany with a total distance of 50 km in water of approximately 16°C within 19 h and 15 min. In 2010 and 2011, he completed in total fourteen 24-h swims in pools where he achieved distances up to 53 km. For this specific cold water swim, he started preparing in January 2012 with open-water swims. The first swim was in January 21 in water of 1.7°C where he achieved 1 km in 32 min. With increasing temperature, he could increase the length of the swim distance, leading to a swim time of 1 km for 28 min in water of 4.2°C and 33 min in water of 4.7°C. In addition to the open-water swims, he trained in a heated (25°C) indoor pool where he completed training units between 4 and 10 km at a mean speed of approximately 2.8 km/h.

### The event

The athlete started on 7 April 2012 at 2:00 a.m. in the harbor of Lindau at the Lake Constance, Germany. He was followed by a large motor boat called ‘Seewolf’ and supported by a little boat called ‘Delfin’. On the Delfin, a crew of three people was close to the swimmer. Two experienced divers were ready for immediate rescue, if needed. On the Seewolf, a medical team with full equipment for reanimation including a defibrillator was in contact with the crew on the Delfin via radio. The support crew on the Delfin provided in regular intervals of 30 to 45 min a standardized carbohydrate gel, the ‘Hammer Gel’ (http://www.hammergel.de), with water containing 61 g of carbohydrates per 100 g of gel as a drink in a small bottle.

### Measurements and calculations

Before the start of the swim, anthropometric characteristics such as body mass, body height, the circumferences of the limbs and the thicknesses of skinfolds at the pectoral, mid-axilla, triceps, subscapular, abdominal, suprailiac, front thigh, and medial calf sites were measured. The circumferences of the limbs as well as all skinfold thicknesses were measured on the right side of the body. Based on these data, body mass index, percent body fat, fat mass, and skeletal muscle mass, using anthropometric methods, were calculated. Body mass was measured using a commercial balance (Beurer BF 15, Beurer, Ulm, Germany) with a precision of 0.1 kg. Body height was determined using a stadiometer with a precision of 1.0 cm. The circumferences of the limbs were measured using a nonelastic tape measure (KaWe CE, Kirchner & Wilhelm GmbH + Co. KG, Asperg, Germany) with a precision of 0.1 cm. The circumference of the upper arm was measured at mid-arm; the circumference of the thigh was taken at mid-thigh, and the circumference of the calf was measured at maximum girth. All skinfold data were obtained using a skinfold caliper (GPM-Hautfaltenmessgerät, Siber & Hegner, Zurich, Switzerland) and recorded to the nearest 0.2 mm. The skinfold measurements were taken once for all skinfold sites. The anatomical sites for the measurements of skinfold thicknesses were pectoral (anterior axillary line), mid-axilla (vertical), triceps (in the middle of the upper arm), subscapular (at the angulus inferior scapulae), abdominal (vertical, right to the navel), suprailiac (at the anterior axillary line), front thigh (mid-thigh), and medial calf (maximum girth). The investigator identified the correct anatomical site using an orientation with finger- and handbreadth from prominent anatomical sites, such as a prominent protuberance or insertion of a tendon. The procedure was performed three times, and the mean of the three measurements was used for the analyses. The available time for taking the skinfold measurements was standardized to ensure reliability. According to Becque et al. [12], readings were performed 4 s after applying the caliper. One trained investigator took all the skinfold measurements, as inter-tester variability is a major source of imprecision in skinfold measurements. Intra- and inter-investigator agreement was assessed from 27 male runners prior to an ultra-marathon, based on measurements taken by two experienced primary care physicians [13]. Intra-class correlation (ICC) within the two investigators was excellent for all anatomical measurement sites and for various summary measurements of skinfold thicknesses. Agreement tended to be higher within than between investigators, but it still reached excellent reliability (ICC > 0.9) for the summary measurements of skinfold thicknesses. ICC for investigator 1 versus investigator 1 and for investigator 2 versus investigator 2 for the single skinfold thicknesses were between 0.98 and 0.99, respectively. For the sum of seven and eight skinfolds, respectively, ICC was 0.99. For the sum of eight skinfolds for investigator 1, bias (i.e., average difference between investigator 1 and investigator 2) was −0.515 mm, and standard deviation of the average difference was 1.492 mm; 95% limits of agreement were between −3.439 and 2.409 mm. Percent body fat was estimated using the anthropometric formula according to Ball et al. [14] for males with percent bodyfat = 0.465 + 0.180 × (*Σ*7SF)−0.0002406 × (*Σ*7SF)^2^ + 0.0661 × (age), where Σ7 SF is the sum of seven skinfold thickness of the pectoralis, axilla, triceps, subscapular, abdomen, suprailiac, and thigh in millimeters and age in years. The predicted residual sum of squares (PRESS) *r*^*2*^ was high (0.90), and the PRESS standard error of estimates (SEE) was excellent (2.2% at the mean) for the equation when applied to a sample of 160 men. Fat mass was estimated using the equations from Stewart and Hannan [15] for male athletes: Fat mass(g) = 331.5 × (abdominal skin − fold thickness) + 356.2 × (thigh skin − fold thickness) + 111.9 × (body mass) – 9, 108. The coefficient of determination was 0.82, and the standard error of the estimate was 1,843 g, which is equivalent to 2.4% for a typical athlete in the sample. Skeletal muscle mass (SMM) was estimated using the formula of Lee et al*.*[16] with SMM = Ht × (0.00744 × CAG^2^ + 0.00088 × CTG^2^ + 0.00441 × CCG^2^) + 2.4 × sex – 0.048 × age + race + 7.8 where Ht = height, CAG = skinfold-corrected upper arm girth, CTG = skinfold-corrected thigh girth, CCG = skinfold-corrected calf girth, sex = 1 for male; age is in years, and race = 0 for white men and 1 for black men. This equation was validated using magnetic resonance imagining (MRI) to determine the skeletal muscle mass. There was a high correlation between the predicted skeletal muscle mass and the MRI-measured skeletal muscle mass (*r*^*2*^ = 0.83, *P* < 0.0001, SEE = 2.9 kg). The correlation between the measured and the predicted SMM difference and the measured SMM was significant (*r*^*2*^ = 0.90, *P* = 0.009). In order to compare skinfold thicknesses with the reported data from Keatinge et al. [6], we measured skinfold thickness over the biceps, at the lower corner of the scapula, at the costal margin below the midpoint of the clavicle, and the abdomen 50 mm below and lateral to the umbilicus.

Temperature was continuously measured as body core temperature in the rectum and as body surface temperature at the left forearm and the right calf using Endotherm® (http://www.endotherm.ch). These thermoelectric probes measure temperatures from −40°C to 85°C with a resolution of 0.0625°C and a precision of 0.1°C. The probes were programmed to take one measurement every 12 s (five measurements per minute) and were applied 10 min before the start of the swim in a heated room of 20°C located around 100 m apart from the start of the swim. The probe in the rectum was inserted using a protective container provided by the manufacturer. At the forearm and the calf, the probe was fixed on a neoprene belt of 3 mm in thickness and 8 cm in breadth at the forearm and 12 cm at the calf, and then fixed with a plastic tape. Energy intake during the swim was estimated using the information on the product supplied by the support crew. Energy expenditure was estimated using a stepwise calculation using body mass, mean velocity, and time spent during performance [17]. The completed distance was continuously recorded on the Seewolf using the global positioning system. Water temperature was measured continuously using the onboard thermometer of the Seewolf. Air temperature was provided by the local weather station (http://www.wetter-lindau.de).

## Results

Table 1 presents the anthropometric characteristics of the swimmer. The mean of the four skinfold thicknesses over biceps, at the lower corner of the scapula, at costal margin below the midpoint of the clavicle, and the abdomen 50 mm below and lateral to the umbilicus was 33.4 mm. The water temperature in the lake varied between 9.8°C and 10.0°C with a mean of 9.9°C during the whole period. Air temperature was 11.3°C at the start and dropped to 9.5°C toward the end of the swim. In the last hour of the swim, some heavy rain was falling and a sharp wind from west was blowing. The swimmer achieved a total distance of 15 km within 6 h 02 min while swimming at a mean speed of 2.48 km/h, equal to 0.69 m/s. After two hours of swimming, he suffered from cramps in the right calf. After two hours and a half, he had problems to urinate. After four hours, he felt very cold and suffered from shivering. During the swim, he expended a total energy of ~2,530 kcal. He consumed a total of ~213 g of carbohydrates with a total energy content of ~510 kcal and an energy deficit of ~2,020 kcal resulted.

**Table 1:** Anthropometric characteristics of the subject

**Characteristic**		**Value**
Age (years)		53
Body mass (kg)		110.5
Body height (m)		1.76
Body mass index (kg/m^2^)		35.7
Circumferences	Upper arm (cm)	36.7
	Thigh (cm)	63.9
	Calf (cm)	43.6
Skinfold thicknesses	Pectoral (mm)	36.4
	Clavicula (mm)	30.2
	Biceps (mm)	11.1
	Axilla (mm)	39.2
	Triceps (mm)	14.2
	Subscapular (mm)	46.2
	Forearm (mm)	18.3
	Abdominal (mm)	46.2
	Suprailiac (mm)	41.6
	Thigh (mm)	44.4
	Calf (mm)	42.6
	Sum (mm)	310.2
Percent body fat (%)		34.9
Estimated fat mass (kg)		34.4
Estimated skeletal muscle mass (kg)		38.1

**Figure 1:**
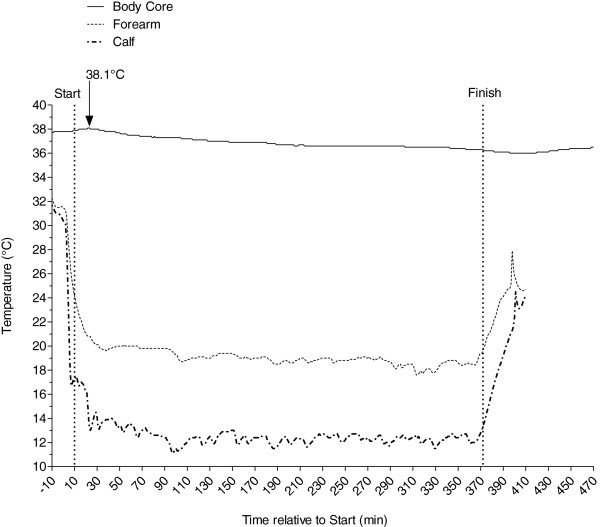
Changes of body core and body surface temperatures during the swim.

Figure 1 shows the development of the temperatures measured in the rectum (body core), at the forearm, and at the calf. At the start of the swim, the body core temperature was at 37.8°C. It increased to a maximum of 38.1°C after approximately 20 min of swimming and then decreased continuously to 36.3°C upon finishing the swim. The lowest body core temperature occurred between 35 and 60 min after finishing the swim with 36.0°C. After 60 min, the body core temperature started to rise continuously, reaching 36.5°C 2 h after getting out of the water. The temperature at the forearm dropped to 19.6°C after 36 min of swimming and decreased to 19.4°C at the end of the swim. The lowest temperature at the forearm was 17.6°C measured approximately 47 min before he stopped swimming. The temperature at the calf dropped to 13.0°C measured after approximately 24 min and decreased to 11.9°C at the end of the swim. The lowest temperature was 11.1°C measured at approximately 108 min after the start. At both the forearm and the calf, the skin temperature continuously increased after the swim.

## Discussion

The most important finding of this investigation was that body core temperature initially increased and then slowly decreased to 36.3°C by the end of the swim. By definition, this cold water swimmer did not suffer from hypothermia [3,8]. Using an equation to predict the survival time of man in cold water with 15 + 7.2/(0.0785 − 0.0034 × water temperature) [18], the swimmer should have died after 2 h and 55 min in water of 9.9°C. Death, however, could also have occurred after the swim. It has been shown that persons evacuated from cold water died in the post-immersion period [19,20]. Ventricular fibrillation is likely during that period, and cardiac failure may follow [21].

### Body core temperature and hypothermia

The normal core body temperature of a healthy, resting adult human being is stated to be between 36.1°C and 37.8°C [22]. Although the body temperature of an individual can vary, a healthy human body can maintain a fairly consistent body temperature between 36.8°C and 37.0°C [23,24]. There are different values for normal temperatures regarding oral, rectal, tympanic, and axillary body temperatures [24]. The range for oral temperature is 33.2°C–38.2°C, for rectal 34.4°C–37.8°C, for tympanic 35.4°C–37.8°C, and for axillary 35.5°C–37.0°C. The range for oral temperature in men and women is 35.7°C–37.7°C and 33.2°C–38.1°C, for rectal 36.7°C–37.5°C and 36.8°C–37.1°C, and for tympanic 35.5°C–37.5 and 35.7°C–37.5°C, respectively [24]. Hypothermia is defined as any body temperature below 35.0°C [4,8,9]. It is subdivided into four different degrees such as mild hypothermia (32°C–35°C), moderate hypothermia (28°C–32°C), severe hypothermia (20°C–28°C), and profound hypothermia at <20°C [25]. Respecting these definitions, our swimmer was able to maintain his body core temperature within the normal range without suffering hypothermia.

### Skinfold thicknesses and body fat

The most likely reason for the finding that our swimmer was able to swim for 6 h and 2 min in water of 9.9°C was his high fat mass [5,26,27], his high body mass index [8], and his experience in open-water ultra-swimming in cold water [7]. In a field study on eight swimmers in the study of Keatinge et al. [6], the swimmers' body core temperatures dropped by 1.94°C after 52.3 min. The mean duration of the swim was related to the mean skinfold thickness of the recorded four skinfold thicknesses. The present swimmer had a mean skinfold thickness of 33.4 mm for the four skinfolds. In contrast to the skinfold thickness of 13.64 mm of the eight swimmers in Keatinge et al. [6], the mean of the four skinfolds was approximately 2.4 times higher in the present swimmer. Following Pugh and Edholm [28], the most important and certainly the clearest answer to the question of whether the heat loss in the Channel swimmers was diminished owing to their body: all the Channel swimmers were fat and many of them were grossly fat. Body fat provided an insulator to reduce heat loss [28]. Heat is produced and stored in the body core and flows down the thermal gradients' esophageal temperature—skin temperature and skin temperature—water temperature [29]. The flow of heat from the core to the skin can be affected by the characteristics of two parallel resistors: one is a fixed resistance which is the layer of body fat, and the other is a variable resistance which represents the peripheral circulation [29].

A relationship between individual skinfold thickness and change in esophageal temperature during submaximal swimming in 18°C and 26°C has been shown [29-31]. Internal temperature changes after 20 min of submaximal swimming were related to water temperature, swimming intensity, and body insulation [29]. Mean skin temperatures were related to both water temperature and water velocity during rest and to water temperature during swimming [29]. Furthermore, the human fat is a better insulator than the human muscle [32]. Fat becomes an effective means of body insulation at high rates of heat flow [33]. This is the situation which prevails during long-distance swimming since swimmers have a high heat production and are immersed in cold water. Under such conditions, each millimeter of subcutaneous body fat will sustain a temperature difference of 1.67°C [33]. The importance of thick skinfolds seems to depend upon the temperature of the water. Pugh et al. [33] reported the skinfold thickness of the Channel swimmers at 15.5°C–18°C and their performance. The winners tended to be less fat than the slower swimmers. Cold tolerance in long-distance swimmers is complex and may involve a factor related to habituation [27,34]. Cold-accustomed swimmers tolerated immersion in cold water better than unaccustomed swimmers [34]. Golden et al. [27] described three swimmers who competed in Lake Windermere. When swimming in the lake, the two cold-accustomed swimmers were faster than the swimmer with less tolerance, although he had a greater mean skinfold. Modern competitive distance swimmers in open water seem to be relatively lean [27].

We placed one thermoelectric probe at the forearm and one thermoelectric probe at the calf to measure the temperature at the surface of the body. We were surprised that we found a difference of approximately 7°C between the surface temperature at the calf and the forearm. The most probable explanation would be the difference in the thickness of the skinfolds at these two sites. At the forearm, the temperature remained rather unchanged at approximately 19°C; at the calf, the temperature remained at approximately 12°C. The skinfold thickness at the forearm was 18.3 mm; at the calf, it was 42.6 mm. The thicker skinfold at the calf site showed obviously a better isolation against the cold water than the thinner skinfold thickness at the forearm site.

The estimated fat mass of 34.4 kg was most probably a very useful isolation towards the cold water [35,36]. Also, a high fat mass may increase buoyancy in water [5]. In a case report with two swimmers in ice-cold water, the swimmer with a sum of eight skinfolds of 139.8 mm was able to swim for 42 min in water of 4°C, whereas the other swimmer with a lower sum of 124.8 mm stopped the swim after 23 min [7]. Petrofsky and Laymon [37] showed that subjects with high body fat changed their deep tissue temperatures much slower than thin subjects when the lower body was immersed in water at 42°C, 37°C, 34°C, 27°C, 24°C, and 0°C for 20 min. Keatinge [35] demonstrated that the fattest man suffered relatively small falls in rectal temperature at both 5°C and 15°C cold water compared with thinner men, and Cannon and Keatinge [38] reported that fat men could reduce their loss of heat more than thin men in the cold. Keatinge [36] showed a linear relationship between rectal temperatures and mean skinfold thickness when subjects were immersed for 30 min in water of 15°C.

Apart from fat mass, a high body mass index may also help humans to stay longer in cold water. Nuckton et al. [8] demonstrated that body mass index correlated with the lowest recorded body core temperature and afterdrop duration when swimming in water of 11.7°C. An important finding was that body core temperature rose to 38.1°C after 20 min of swimming, and the lowest body core temperature was found between 35 and 60 min after finishing the swim with 36.0°C. Upon finishing the swim, the body core temperature was at 36.3°C. Thus, the decrease by 0.3°C corresponds to an afterdrop, which is defined as continued cooling following removal from cold stress [8]. The occurrence of an afterdrop appears not to depend upon the existence of a circulation but may be explained by a thermal conduction mechanism [39]. Afterdrop seems to be more frequent than hypothermia in cold water swimmers. Nuckton et al. [8] investigated 11 subjects in the New Year’s Day Alcatraz Swim. While 5 of the 11 subjects suffered from hypothermia, afterdrop was seen in 10 of the 11 subjects.

## Conclusions

The present case report shows that it is possible to swim for 6 h in water of 9.9°C without signs of hypothermia. The high body mass index, high body fat, previous experience with cold habituation, and specific preparation of the swimmer are the most probable explanations for these findings.

## Consent

Written informed consent was obtained from the athlete for publication of this case report. A copy of the written informed consent is available for review by the Editor-in-Chief of this journal.

## Competing interests

The authors declare that they have no competing interests.

## Authors’ contributions

CAR collected the data and drafted the manuscript. BK and TR helped draft the manuscript. All authors read and approved the final manuscript.
